# “ReachOut”: Pilot Evaluation of a Help-Seeking Intervention for Common Mental Health Concerns Among Distressed Non-Treatment-Seeking Young Adults

**DOI:** 10.7759/cureus.54324

**Published:** 2024-02-16

**Authors:** Prachi Sanghvi, Seema Mehrotra, Manoj Sharma

**Affiliations:** 1 Clinical Psychology, National Institute of Mental Health and Neurosciences, Bengaluru, IND

**Keywords:** common mental health concerns, young adult, technology, help-seeking behavior, help-seeking inclination, help-seeking intervention

## Abstract

Purpose

There is a pressing need for interventions with the potential for scalability to enhance help-seeking inclination and behavior among individuals experiencing common mental health concerns. These interventions are important for addressing the widespread treatment gap. This study aimed to test the effectiveness, feasibility, and acceptability of a newly developed simple technology-based multi-component help-seeking intervention ("ReachOut") for common mental health concerns among distressed, non-treatment-seeking young adults.

Methods

"ReachOut" was delivered to 172 young adults aged 20-35 years, scoring above the cut-off on the Kessler Psychological Distress scale. Effectiveness was studied using a single-group short-term prospective study design to examine changes in help-seeking barriers, inclination, and behavior. We assessed intervention feasibility in terms of demand, implementation, practicality, and limited efficacy and acceptability was determined based on the rate of participation consent, the extent of pro-active initiation of contact with the facilitator during the intervention, feedback obtained on various "ReachOut" components and ratings on the likelihood of recommending the intervention to a person in distress.

Results

Significant reductions in the mean barriers and improvement in mean help-seeking inclination from mental health professionals (MHPs) were observed on the Friedman test from baseline to the two-month follow-up period after the intervention. Thirty-eight percent of participants (N=41) reported seeking help from MHPs by two-month follow-up. Feedback from participants, assessments, and observations indicated that "ReachOut" was feasible and acceptable among the target sample.

Conclusions

The study provides preliminary evidence of the effectiveness, feasibility, and acceptability of the help-seeking intervention "ReachOut" in reducing barriers and improving help-seeking inclination and behavior for common mental health concerns among distressed non-treatment-seeking young adults.

## Introduction

Help-seeking comprises formal professional help as well as seeking help from informal sources like family and friends. Young adults, who form the largest part of the productive segment of society, bear the maximum burden of mental disorders [1]. High treatment gap [2] and hesitation to seek professional help for common mental health concerns have been observed among young adults for common mental health concerns.

The reasons for not seeking professional help among this group have been examined in various studies [3]. These include low mental health awareness, stigma, preference for informal sources of help, instrumental barriers, etc. To tackle these barriers, help-seeking interventions for mental health are delivered that generally focus on changing attitudes, intentions, and behavior. However, universal help-seeking interventions directed at unselected general community samples lack the scope for contextualized, individualized, and detailed inputs to help individuals with distress [4]. Hence, there is a need for indicated interventions targeting distressed-non-treatment seekers, which encourage professional help-seeking, leading to early detection and initiation of treatment.

Various behavior change interventions for help-seeking in mental health, including those with common mental disorders among young adults [5], have been developed and tested to reduce help-seeking barriers, improve self-identification of symptoms, and facilitate early treatment. However, several of them have not considered the specific barriers of this target group, including a preference for self-reliance and informal sources, a tendency towards normalization of distress, and concerns about treatment effectiveness. Most of them include one-to-two components mainly focused on help-seeking attitudes and inclinations. The need for help-seeking interventions with multiple components and personalized elements targeting help-seeking behavior has been highlighted.

Numerous help-seeking interventions across the globe have been delivered through various modalities, including face-to-face discussions and telephonic and online modes of delivery, including WhatsApp, email, use of mobile phones, and multimedia [6]. These technologies providing e-mental health services are found to be the preferred mode of communication among young adults, especially in the Indian context [7]. Technology-based interventions can not only augment the delivery of mainstream treatment services but also serve as an important pathway to reach out to distressed young adults in the community who are not yet availing treatment. Literature reviews highlight a dearth of research on interventions to enhance help-seeking among distressed young adults in the country [6,8]. This study aimed to address the aforementioned gaps in the literature by assessing the effectiveness, feasibility, and acceptability of "ReachOut," a simple technology-based, multi-component help-seeking intervention for common mental health concerns among distressed non-treatment-seeking young adults in the urban Indian context.

## Materials and methods

Study design and objectives

A single-group short-term prospective study design was employed to evaluate the utility of help-seeking intervention in reducing barriers to professional help-seeking and improving help-seeking inclination and behavior from baseline to follow-up assessment (primary outcome variables). The study also assessed changes in help-negation, inclination to seek help from non-professional and professional sources in general, and likelihood to consult a mental health professional (MHP) in the next two months (secondary outcome variables).

Feasibility was ascertained using two frameworks of Bowen et al. [9] and Orsmond and Cohn [10]. It was evaluated in terms of demand, implementation, practicality, and limited efficacy of the help-seeking intervention. Acceptability of the intervention was determined based on the rate of obtaining participation consent, the extent of pro-active initiation of contact with the facilitator during the intervention, feedback obtained on various "ReachOut" components, and other parameters, including ratings on the likelihood of recommending the intervention to a person in distress.

Participants and sampling method

Distressed non-treatment-seeking young adults between the ages of 20 and 35 years with at least 12 years of formal education and a self-report of the current experience of psychological distress for a minimum of two weeks, scoring above the cut-off for distress on the Kessler Psychological Distress Scale (K10) [11], not seeking professional help for their current distress, having working knowledge of the English language, and having access to and comfort in using the internet were recruited for the present study.

Being a hard-to-access population, snowball sampling was used in addition to purposive sampling. A recruitment announcement was used to invite young adults to participate if they were experiencing distress (mood/anxiety symptoms) and not availing of professional help. This was disseminated through various modalities like social media platforms and word-of-mouth publicity.

Sample size estimation

**Figure 1:**
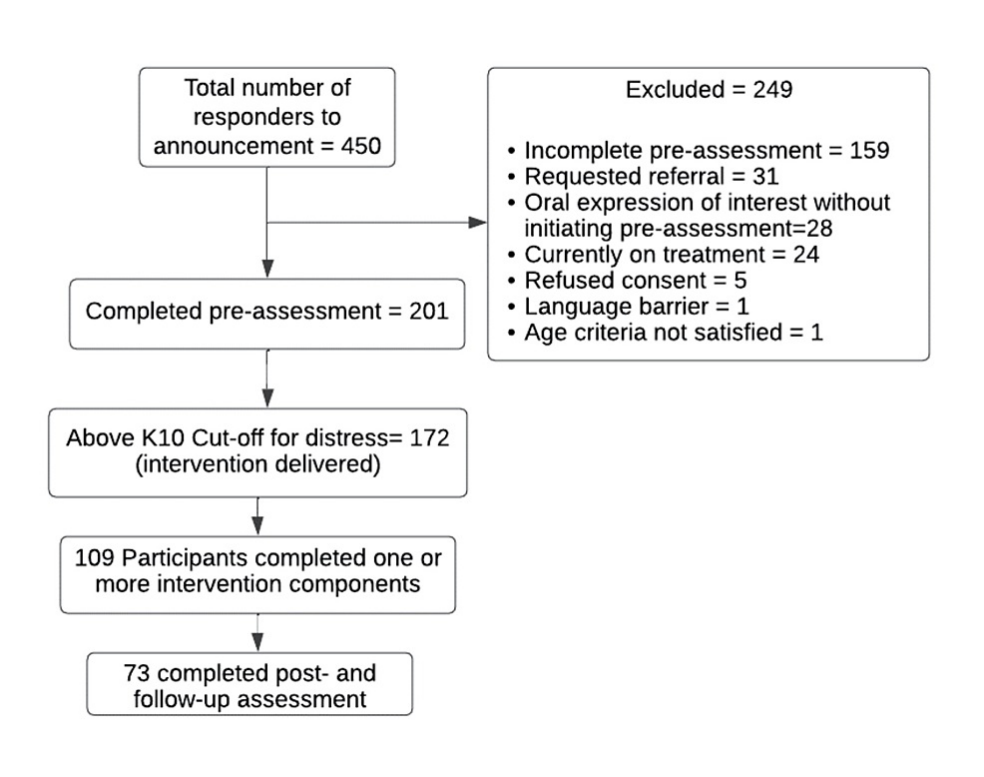
Flow of participants throughout the study K10: Kessler Psychological Distress Scale

The sample size was determined using the G*Power software version 3.1.9.7 (Universität Düsseldorf, Düsseldorf, Germany). Due to high variability in terms of the duration and nature of the help-seeking interventions and the nature of the samples studied, data from previous studies could not be used for sample size estimation. Based on the research design, anticipating help-seeking behavior for 80% of the sample during the help-seeking intervention period and considering a precision of 6% for a 95% confidence level, the minimum sample size required was 171. The flow of the participants throughout the study process has been described in Figure 1.

"ReachOut": the help-seeking intervention

"ReachOut" was developed by the authors as a WhatsApp-based help-seeking intervention for distressed young adults to be delivered by healthcare professionals. It is based on the preferences of young adults elicited through an exploratory study and literature reviews [6,8,12]. The exploratory study helped in understanding barriers and enablers in distressed young adults and their perspectives on potential content and modes of delivery of an intervention to enhance professional help-seeking. Eight core intervention components and one optional component were developed which were built upon relevant theoretical frameworks [13,14] and a priori guidelines [15]. These included personalized feedback, recognition of common mental health concerns, overcoming barriers to consulting MHPs, motivational interviewing, facilitating support mobilization from a nominated significant other for professional help-seeking (optional), indirect social contact with mental health service consumers, socialization to the consultation process, experts’ views on mental health consultation, and co-ordinated referral. The intervention components are delivered weekly in a spaced-out fashion over a six to eight-week period. The development and content validation of "ReachOut" have been described elsewhere [15]. These intervention components have been postulated to enhance awareness of common mental health problems, the utility of self-help and support of significant others, and increase the skills to understand when it may be appropriate to step up to professional help-seeking.

Tools

Baseline Assessment

Using the SurveyMonkey platform, the baseline assessment elicited socio-demographic details and items to capture current perceived distress severity, duration, functioning difficulty, overall usefulness of self-help methods, and past treatment history. An additional item on the likelihood of consulting in the next two months (Likert-type) was also added. Furthermore, standardized measures, including K10 for psychological distress [11] and the General Help-Seeking Questionnaire (GHSQ) [16] for the inclination to seek help were used. The barriers to seeking professional help for the Mental Health Scale were also administered during the baseline assessment. It was developed based on the exploratory study findings [12] and consisted of 28 items that captured barriers to consult MHP for current distress. Each item was rated on a five-point Likert scale from "not at all" to "to a great extent." The sub-scales include distress perception, stigma, apprehensions about service utilization, and instrumental barriers. A higher total or sub-scale score indicated more number and strength of the barriers. The overall reliability of the scale was found to be good (α=0.89), with sub-scale reliabilities ranging from α=0.70 to α=0.83.

Assessment During the Help-Seeking Intervention

During the delivery of the "ReachOut" intervention, two items were used at three time points to assess if help was sought from an MHP (yes/no) and inclination to consult an MHP (seven-point Likert scale) if help-seeking behavior had not occurred.

Post-assessment

Once the delivery of intervention components was completed or sought professional help at any time during the intervention, a repeat online survey was conducted on the same platform. This included K10, GHSQ, and barriers to seeking professional help for the Mental Health Scale. Items on help-seeking behavior from MHP and the likelihood of seeking professional help in the next two months (if help was not sought) were also included. Additionally, feedback probes were used to examine the acceptability of "ReachOut."

Follow-Up Assessment

The first follow-up assessment was conducted one month after the post-assessment using the same online survey to elicit barriers to consult MHP, help-seeking inclination, and behavior (F1). Participants who had not consulted MHP were approached over the phone one month after F1 to inquire about their help-seeking behavior as part of the second follow-up (F2).

Procedure

**Figure 2:**
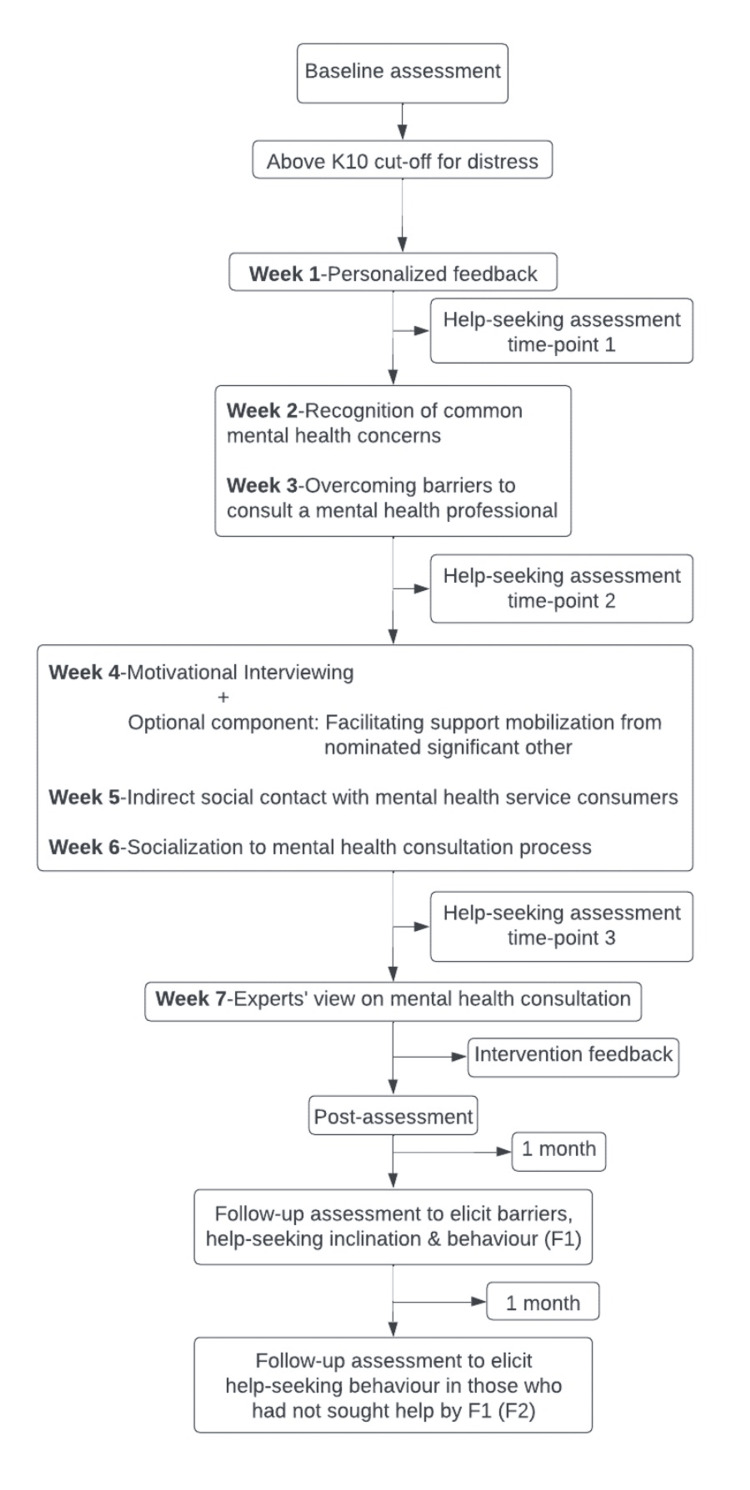
Structure of the help-seeking intervention ("ReachOut") K10: Kessler Psychological Distress Scale

The study was initiated after approval from the Institute Ethics Committee (approval no.: NO.NIMH/DO/IEC (BEH. Sc. DIV)/2019). The intervention trial was registered in the International Standard Randomised Controlled Trial Number (ISRCTN) registry (ISRCTN14504454). Figure 2 summarizes the intervention procedure. PS was the intervention facilitator in the present study. A baseline assessment was conducted to assess participants’ distress levels. Those with scores above the cut-off on K10 were invited to engage with "ReachOut" (N=172). The intervention components were delivered in a spaced-out fashion over six to eight weeks in participants’ preferred communication mode, mainly WhatsApp (or email if preferred). These components were delivered in audio, video, or poster format and included a single 30-45 minute audio session for motivational interviewing and support mobilization from the nominated significant other. The motivational interviewing session focused on addressing specific barriers for the participants to seek professional help. This was attempted through empathizing with their distress, eliciting desired goals, the discrepancy between their current behaviors and the goals, and discussing the potential benefits of professional help-seeking and ways of negotiating barriers to seeking such help.

Each component was delivered via Google form as an attachment with one-to-two content-related questions to verify participants’ engagement with the components. Participants were also approached individually to confirm engagement for each component if not confirmed via the Google forms. Each participant was given a unique participant ID that they were supposed to enter in the Google form while engaging with each intervention component. This was to ensure that no personally identifying information was required to be mentioned to maintain confidentiality and anonymity. A range of five to nine days with an average of seven days to deliver each component was decided depending on when the participant engaged with the component. A maximum of two reminders were sent for each component. If any participant reported seeking professional help at any point during the intervention, it was considered an end-point for them. A telephonic feedback on "ReachOut" was conducted with the participants within one week of completion.

Data analyses

Data were analyzed using the IBM SPSS Statistics for Windows, Version 20 (Released 2011; IBM Corp., Armonk, New York, United States). Frequency, percentage, mean, and standard deviation were used as part of the descriptive statistics. Changes in the primary and secondary outcome variables during pre-, post-, and follow-up assessment were examined using repeated measures ANOVA/Friedman test. Additionally, effect sizes were calculated.

## Results

Sample characteristics and distress profile

Table 1 describes the socio-demographic characteristics of the intervention sample, and Table 2 describes the baseline clinical characteristics. Perceived distress severity was rated as moderate, and functional impairment was rated as high by 41% and 40% of the participants (N=70), respectively. On the whole, self-help methods were rated as slightly helpful in managing distress by about 40% of the participants (N=68). The highest help-seeking inclination across all sources on GHSQ was found to be for friends/colleagues (58%, N=99). Preference for self-reliance, financial constraints, and normalization of distress were top barriers to seeking professional help for current distress.

**Table 1:** Socio-demographic characteristics of the intervention sample (N=172) The data has been represented as N, %.

Variable	Group	Frequency	Percentage
Age x̄ (SD)=26.39 (3.86)	20-24	64	37.20
	25-29	73	42.44
	30-35	35	20.36
Gender	Women	119	69.2
	Men	53	30.8
Education	Up to 12th grade	4	2.3
	Graduation/diploma	65	37.8
	Post-graduation and above	103	59.9
Occupation	Salaried employee	78	45.3
	Student	52	30.2
	Looking for a job	24	14
	Self-employed	16	9.3
	Homemaker	2	1.2
Individual annual income	Not applicable	58	33.7
	Below 19,000	6	3.5
	20,000-50,000	15	8.7
	50,000-1 lakh	11	6.4
	1 lakh and above	56	32.6
	Prefer not to disclose	26	15.1
Marital status	Unmarried	134	77.9
	Married	38	22.1
Religion	Hindu	100	58.1
	Christian	33	19.2
	Muslim	11	6.4
	Do not follow any religion	26	15.1
	Other	2	1.2
Current living arrangement	With family	121	70.3
	Living alone	18	10.5
	Hostel	18	10.5
	With flatmates	15	8.8
City type	Metropolitan	94	54.7
	Urban	56	32.6
	Semi-urban	19	11.0
	Rural	1	0.6
	Missing	2	1.2

**Table 2:** Baseline clinical characteristics of the intervention sample (N=172) The data has been represented as N, %. K10: Kessler Psychological Distress Scale

Variable	Group	Frequency	Percentage
Self-reported symptoms	Anxiety	39	22.7
	Depressive	41	23.8
	Both	92	53.5
Symptom duration	Less than 2 weeks	6	3.5
	About weeks	2	1.2
	2 weeks to 1 month	21	12.2
	1-3 months	31	18
	3-6 months	27	15.7
	More than 6 months	85	49.4
Perceived distress severity	Minimal	15	8.7
	Mild	34	19.8
	Moderate	70	40.7
	Quite very severe	52	30.8
Distress severity on K10 Mean (SD) K10 score=31.1 (6.43)	Mild	27	13.43
	Moderate	45	22.39
	Severe	100	49.75
Self-reported functioning impairment	Not at all	8	4.7
	Slightly	31	18
	To some extent	63	36.6
	To a great extent	53	30.8
	Extremely	17	9.9
Consultation for mental health at any time in the past	Consulted	57	33.1
	Not consulted	115	66.9
Encouragement from significant other for professional help-seeking for current mental health concern	Yes	79	45.9
	No	77	44.8
	Can’t say	16	9.3
Perceived likelihood of professional help-seeking in the next two months	1 (extremely unlikely)	26	15.1
	2	12	7
	3	11	6.4
	4 (unsure)	73	42.4
	5	15	8.7
	6	15	8.7
	7 (extremely likely)	20	11.6
Five most endorsed barriers	Wanting to solve the problem on my own	76	44.2
	Financial constraints	73	42.4
	My current problem is just a passing phase	70	40.7
	I won't be able to clearly tell what's troubling me	69	40.1
	My current problem is not severe	59	34.3

Engagement with "ReachOut"

Of the 172 participants, 109 engaged (63.4%) with at least one "ReachOut" component as verified via Google Forms or individual confirmation. The engagement with each intervention component ranged from 28% to 63%, as verified (N=30-109). In addition, 19% of the participants (N=21) availed of the optional component aimed at mobilizing the support of significant others to seek professional help. Most of the nominated significant others were friends/partners. Table 3 describes the number of each intervention component completed by the participants as verified. Reasons for missed engagement with one or more "ReachOut" components could be elicited from 40% of participants (N=51), wherein a majority reported practical constraints.

**Table 3:** Number of components engaged with by intervention sample as verified (N=109) The data has been represented as N, %.

No. of intervention components completed as verified	Frequency	Percentage
1	8	7.34
2	22	20.18
3	13	11.93
4	12	11.01
5	7	6.42
6	4	3.67
7	43	39.45

Changes in outcome variables

Significant decrease in overall barriers and their subscales, help negation, and increase in help-seeking inclination from professional sources, specifically MHPs and likelihood to consult a professional in the next two months, were noted at post-assessment, and gains were maintained/strengthened at follow-up on most variables (Table 4). Medium effect sizes were noted for most variables (Table 5).

**Table 4:** Changes in key help-seeking variables across intervention time points (N=63) ^1^Non-professional sources: friend/colleague, partner/spouse, parent, other family member/relative; ^2^Professional sources: helpline, general physician, other health professionals, mental health professionals; ^+^Repeated measures ANOVA; ^*^One-tailed test of significance HS: Help-seeking; MHP: Mental health professional; FU: Follow-up; NS: Not significant The data has been represented as mean±SD, ꭓ^2^/F (Friedman test/ANOVA). p<0.05 was considered significant.

Variable	Mean (SD)			ꭓ^2^/F (p)	Pair-wise comparison (p)		
	Pre	Post	Follow-up		Pre-post	Pre-FU	Post-FU
Total barrier score	36.87 (19.4)	21.08 (14.27)	18.51 (11.57)	50.58 (<0.001)*	<0.001	<0.001	0.02
Distress perception subscale	8.84 (5.3)	5.6 (4.31)	4.95 (3.49)	33.79 (<0.001)*	<0.001	<0.001	NS
Stigma subscale	6.44 (5.99)	2.9 (2.91)	2.9 (3.08)	29.34 (<0.001)*	<0.001	<0.001	NS
Apprehensions about service utilization subscale	13.29 (8.95)	7.29 (6.99)	6.56 (5.53)	37.77 (<0.001)*	<0.001	<0.001	NS
Instrumental barriers subscale	8.3 (4.8)	5.27 (3.94)	4.1 (3.64)	35.43 (<0.001)*	<0.001	<0.001	0.002
Help negation	3.51 (1.99)	2.62 (1.9)	2.54 (1.83)	22.84 (<0.001)	<0.001	<0.001	NS
Mean HS inclination from non-professional sources^1^	3.33 (1.18)	3.49 (1.15)	3.32 (1.32)	1.208^+^ (NS)	-	-	-
Mean HS inclination from professional sources^2^	2.9 (1.14)	3.78 (1.18)	3.42 (1.16)	20.44^+^ (<0.001)	<0.001	0.007	0.001
HS inclination from MHP	4.41 (1.8)	5.62 (1.31)	5.54 (1.34)	32.38 (<0.001)*	<0.001	<0.001	NS
Likelihood to consult MHP in the next two months (N=36)	3.28 (1.89)	4.56 (1.68)	4.28 (1.63)	16.294 (<0.001)	<0.001	<0.001	NS

**Table 5:** Effect sizes of key help-seeking variables from baseline to post-assessment (N=73) ^1^Non-professional sources: friend/colleague, partner/spouse, parent, other family member/relative; ^2^Professional sources: helpline, general physician, other health professionals, mental health professionals; ^3^N=53 after removing participants who sought professional help; ^†^Paired t-test HS: Help-seeking; MHP: Mental health professional The data has been represented as mean±SD, t/z, and r. p<0.05 was considered significant.

Variable	Mean (SD)		t/z	p	Effect size (r)
	Pre	Post			
Total barrier scale score	36.19 (19.05)	22.6 (16.11)	5.11	<0.001	0.42
Distress perception subscale	8.82 (5.21)	5.95 (4.3)	4.56	<0.001	0.38
Stigma subscale	6.22 (5.87)	3.55 (4.32)	4.02	<0.001	0.33
Apprehensions about service-utilization subscale	13.19 (8.67)	7.93 (7.49)	4.72	<0.001	0.39
Instrumental barriers subscale	7.96 (4.95)	5.32 (4.09)	3.99	<0.001	0.33
Help negation	3.63 (2.06)	2.68 (1.87)	4.057	<0.001	0.34
Mean HS inclination from non-professional sources^1^	3.27 (1.22)	3.55 (1.19)	1.96^†^	0.054	0.22
Mean HS inclination from professional sources^2^	2.82 (1.15)	3.76 (1.13)	6.63^†^	<0.001	0.62
HS inclination from MHP	4.3 (1.87)	5.58 (1.32)	5.40	<0.001	0.45
Likelihood of consulting MHP in the next two months^3^	3.4 (1.74)	4.66 (1.68)	4.28	<0.001	0.35

Help-seeking behavior

The status of professional help-seeking was available for 109 participants by the end of the second follow-up, as assessed at three time points during the intervention (T1, T2, T3; Figure 2), at the post, and at two follow-up time points (Table 6). Forty-one participants (37.6%) reported seeking professional help by the end of the study. This is higher than the 12% rate of treatment seekers for common mental disorders reported in the Indian population [17] (z=8.22, p<0.01). After the second follow-up, six participants additionally sought professional help but were not included in the analysis.

**Table 6:** Professional help sought by intervention participants at various time points (N=109) The data has been represented as cumulative %.

Help-seeking assessment time-points	Professional help sought frequency (cumulative %)
By T1	0 (0)
By T2	8 (7.3)
By T3	17 (15.6)
By post-assessment	22 (20.2)
By follow-up assessment 1	34 (31.2)
By follow-up assessment 2	41 (37.6)

Feasibility

The feasibility of the intervention was ascertained using specific criteria of the two frameworks [9,10] as described in Table 7.

**Table 7:** Feasibility parameters of the help-seeking intervention D-NTS: Distressed non-treatment seekers; MHP: Mental health professional

Parameters	Comments
Demand	Time taken to reach the pre-defined sample from the date of recruitment announcement: five months; Rate of refusal of consent: 1.11%; Rate of engagement with intervention: 63% of those who completed baseline assessments. Almost 40% of participants engaged with all intervention components
Implementation	Recruitment announcement: circulation through various modes; Sampling: purposive, snowball, and word-of-mouth publicity; Delivery modes incorporated as per the preference of the sample; Most appealing feature: intervention structure (systematic/spaced-out format and the ability to reach out to the D-NTS) as per feedback by 72% of participants; Positive feedback on the adequacy of engagement with components once a week and with overall intervention duration
Practicality	Flexibility for delivery formats; Intervention delivery: range of 5-9 days spacing for each component
Limited efficacy	Significant changes in key outcome variables 38% of participants had sought help from MHP for their current distress by follow-up

Acceptability

During the intervention, participants proactively contacted the facilitator to clarify help-seeking-related concerns and seek referral-related guidance. Table 8 provides the participants' rating for the usefulness of each intervention component rated on a 10-point Likert scale.

**Table 8:** Usefulness rating of the intervention components Rating: 1: Not at all useful, 10: Extremely useful MHP: Mental health professional The total number differs in each component depending on whether the participants went through the component or had already sought help before reaching the component. The data has been represented as N, %, and mean±SD.

Component	Rating (frequency/%)				x̄ (SD)
	1	1.5-4.5	5-7.5	8-10	
Personalized feedback (N=69)	1 (1.45)	1 (1.45)	16 (23.19)	51 (73.91)	6.69 (3.11)
Recognition of common mental health concerns (N=63)	1 (1.59)	4 (6.35)	11 (17.46)	47 (74.6)	5.64 (2.95)
Overcoming barriers to consult MHP (N=62)	-	4 (6.45)	12 (19.35)	46 (74.19)	6.89 (2.43)
Motivational interviewing (N=59)	-	-	2 (3.39)	57 (96.61)	8.67 (1.08)
Indirect social contact with mental health service consumers (N=51)	1 (1.96)	3 (5.88)	4 (7.84)	43 (84.31)	6.29 (2.84)
Socialization to consultation process (N=46)	1 (2.17)	2 (4.35)	5 (10.87)	38 (82.61)	6.06 (3.32)
Experts’ view on mental health consultation (N=45)	-	2 (4.44)	7 (15.56)	36 (80)	6.5 (2.74)
Facilitating support mobilization from nominated significant other (N=20)	-	1 (5)	3 (15)	16 (80)	7.43 (2.59)
Co-ordinated referral: researcher’s availability for consultation guidance/clarification (N=68)	-	-	2 (2.94)	66 (97.06)	8.50 (1.29)

Table 9 describes participants' ratings on the usefulness of various aspects of "ReachOut."

**Table 9:** Participants’ rating on the usefulness of various aspects of "ReachOut" (N=69) * Two participants did not go through enough components to provide the rating. The data has been represented as N, %, and mean±SD.

Item	Rating (frequency/%)				x̄ (SD)
	1	1.5-4.5	5-7.5	8-10	
Comprehensive coverage*	-	-	12 (17.91)	55 (82.09)	8 (1.37)
Convenience of participation	-	-	6 (8.7)	63 (91.3)	8.33 (1.08)
Potential usefulness in motivating young adults to consult	-	-	15 (21.74)	54 (78.26)	7.5 (1.56)
Likelihood of recommending intervention to distressed others	-	-	10 (14.49)	59 (85.51)	7.88 (1.75)
Overall usefulness of the intervention	-	-	9 (13.04)	60 (86.96)	8.29 (1.41)

## Discussion

Socio-demographic characteristics and distress profile

The present study examined the effectiveness of "ReachOut," a newly developed help-seeking intervention among distressed young adults. Distress severity on K10 for most participants was above the cut-off as intended, showing the success of the recruitment method. While most participants perceived their distress as moderate, their K10 scores reflected severe levels of distress. These differences between self-perceived distress severity versus scores on standardized psychological distress measures may be attributable to a lack of self-awareness, a tendency towards normalization of distress and poor mental health literacy [3], as also seen in the barriers to seeking help in this sample. Preference for self-reliance and normalization of distress emerged as salient barriers to professional help-seeking. Men accounted for only one-third of the total sample. Internalized masculine norms negatively affect men’s help-seeking behavior, primarily shaped by sociocultural factors [18], and may have influenced men’s interest in participating in the present study. Preference for self-reliance and normalization of distress are also critical barriers in professional help-seeking. This trend echoes the need for de-stigmatization of mental health and interventions to improve help-seeking among distressed young adults [19].

Baseline findings on preference for informal sources of help-seeking, particularly one’s social network, have been noted as a key source of mental health support among young adults in the Indian and global literature [20]. Comparatively lower reliance on informal sources other than friends could reflect a lower preference for other sources of informal support in the target age group or lower perceived availability of support sources. This points to the need for sensitizing informal networks in the lives of young adults to build trusting relationships that provide the freedom to share one’s mental health concerns.

Engagement with "ReachOut"

"ReachOut" spanned over six to eight weeks, but the occurrence of help-seeking behavior at any point during this period was considered the end-point for the given participant. Generally, the duration of help-seeking interventions found in literature ranged from 40 minutes to 6 weeks, consisting of one to two modules [6]. The engagement rate for various "ReachOut" components, as verified, ranged between 28% and 63%. These rates are impressive for this minimally guided intervention aimed at improving help-seeking, which did not involve direct, verbal interaction with the facilitator except during the delivery of one/two components. Studies have revealed a wide range of engagement rates in online mental health interventions [21,22]. Additionally, engagement rates across studies are not easily comparable due to differences in the nature of the target population, nature of intervention, target variables, etc. Engagement can become even more challenging when a technology-based intervention targets distressed non-treatment seekers with the aim of improving help-seeking.

Changes in outcome variables

Significant changes were noted across time points for all primary and secondary outcome variables except help-seeking inclination from non-professional sources. The intervention in the present study aimed to reduce barriers to professional help-seeking by promoting favorable attitudes towards mental health and the components were designed to address the same. This reduction may be attributed to improved knowledge about mental health and treatment processes, receiving objective and yet non-threatening feedback about one’s level of distress and coping, normalization of help-seeking, learning about advantages of professional help-seeking through experiential accounts of others, and direct and individualized inputs provided through motivational interviewing session. It could also be due to opportunities to openly discuss mental health issues as part of the study and with participants’ significant others, raise concerns, and clarify doubts regarding the same with the facilitator. There is a severe dearth of help-seeking intervention studies assessing change in barriers among young adults. The results of the present study demonstrate changes in all the barrier subscales.

In the present study, there was also a significant reduction in help negation over time. It is possible that participants had a shift in perception of their distress from being "normal distress" to being "real distress" [14], which in turn could have led to help-seeking. Help negation could also be understood in the light of a high preference for self-reliance in dealing with one’s mental health concerns, noted as one of the most endorsed barriers at baseline. It has been identified as one of the key barriers to seeking professional help among young adults in the Indian setting as well as globally [3]. However, self-reliance can also contribute to resilience, which can positively affect health care and recovery [23]. Therefore, the present intervention focused on minimizing the need to depend "exclusively" on self-reliance and utilizing various resources for dealing with distress [24,25], which could have contributed to reduced help negation.

There was no significant change in help-seeking inclination from non-professional sources. Various factors could have influenced these findings, including that this score represented an average across different non-professional sources. The inclination to seek help from friends was the highest, whereas the inclination to seek help from parent(s)/relatives was lower. At baseline, almost half of the participants reported not receiving encouragement from their significant others to consult MHPs for their concerns. Moreover, only 19% availed the optional component of support mobilization from significant others and most nominated friends/partners. Collectivist cultures promote an interdependent view of the self where the self is interrelated and obligated to significant others. Because of the heightened awareness and sensitivity to relational consequences, individuals with interdependent self‐views may adopt greater emotion control values and suppress emotion to preserve social harmony [26]. In the Indian context, a family-based decision-making process for help-seeking is common [8]. However, disclosing to the family about one's mental health concerns will likely depend on anticipated reactions and impact on the family. The ambivalence of wanting to express emotions and fearing the consequences of such expressions can be associated with poor psychological health [27]. Being in a collectivist culture like India could create an ambivalent state. One might wish to seek support from loved ones in getting professional help, but at the same time, one might fear burdening them with their problems and worry about upsetting them by going against their preferences. Therefore, there is a need for further research on approaches that can help equip informal support providers, mainly family members of distressed youth and young adults, with the necessary knowledge and skills to encourage appropriate professional help-seeking among their significant others.

An increase in help-seeking inclination from professional sources was significant from baseline to post-assessment with a large effect size and maintained at follow-up. Specifically, there was an increased inclination for MHP from whom the participants eventually sought help, which could explain this finding. Identifying one’s symptoms as signs of a mental health concern leads to improved help-seeking inclination [28], which is what the initial intervention components aimed to achieve. As a corollary, there was a significant change in the likelihood of consulting MHP over the next two months. The intention of the participants to seek help strengthened as the intervention progressed and concomitantly improved their perceived likelihood of seeking professional help in the near future.

Help-seeking behavior

Although increased inclination is essential to initiate a new behavior, improved help-seeking inclination does not necessarily translate to help-seeking behavior [29], resulting in an intention-behavior gap. Previous intervention studies have mainly examined and targeted improvement in help-seeking inclination [30], while the present study attempted to address and asses both inclination and behavior. By the end of the present study, 38% of the participants had sought help from MHP. The help-seeking behavior has ranged from 9.5% to 50% in various help-seeking intervention studies [24,25]. Moreover, the help-seeking behavior rate observed following the "ReachOut" intervention on a sample of participants who were non-treatment seekers, to begin with, is significantly higher than the national help-seeking rate for common mental health disorders reported in India [17]. This, in turn, adds to the meaningfulness of the findings of this pilot evaluation of the "ReachOut" intervention to improve professional help-seeking.

Not all the participants completed the intervention in the present study before seeking professional help and thus appear to require varied dosages of the intervention. This dosage could depend on how much the person might need to be nudged to propel the action, which may vary depending on the stage of change they are at [31].

Observations on feasibility and acceptability

Demand for the intervention was adjudged to be satisfactory, surmised based on efficient and successful recruitment of the target sample, about two-thirds of the participants engaging in at least one intervention component, with almost 40% completing all seven core components and more than three-fourths of them reporting the intervention to be highly useful for the target group. "ReachOut" could be carried out as planned, which included utilizing multiple approaches for recruitment, delivery of the components at pre-determined intervals through modalities as preferred by the participants, and obtaining post- and follow-up assessment data via online/telephonic modes.

The practicality aspect of the feasibility framework was reflected in the flexibility of the intervention with respect to the mode of component delivery and the spacing between the intervention components. "ReachOut" was planned in a way that participants could connect with the facilitator at any point during the study for any clarification or guidance related to help-seeking. The limited efficacy of "ReachOut" was evident in the significant changes found in the outcome variables. Significant gains in help-seeking are encouraging because the high prevalence of common mental health problems and hesitation to seek professional help increase the treatment gap and lead to increased disease burden and disability [1,2]. The findings reported on feasibility are highly encouraging and suggest that "ReachOut" shows promise as a scalable intervention.

Participants’ feedback on the intervention indicated high receptivity and 86% were highly likely to recommend the intervention to others in distress. The use of theoretical frameworks [13,14] which focus on help-seeking in distressed young adults, insights from exploratory phase data [12], youth-friendly format and content of the help-seeking intervention, as well as the use of simple technology as a mode of delivery may have contributed to the acceptability of the intervention. Feedback indicated that the intervention's design and presentation were well-received, increasing self-awareness and motivating them to seek professional help.

These initial findings suggest that delivering a simple technology-based multi-component help-seeking intervention targeted at urban distressed non-treatment young adults in a low-resource setting was feasible and acceptable.

Limitations

A causal effect on the outcome variables could not be established since the study was only a pilot evaluation and had no control group. Non-responders to the intervention comprised one-third of the participants. There was a reduction in the sample size by follow-up assessment due to attrition, and hence, the findings need to be treated as preliminary, though the attrition is not surprising given the nature of the study. Men participants were underrepresented in the sample. The follow-up period may not have been sufficient for some participants needing more time to exhibit help-seeking behavior after exposure to the intervention. Although supplementary probes were used to understand the nature of help sought, help-seeking behavior was assessed solely based on the self-report of participants, which could be another limitation of the study. The present study mainly focused on common mental health concerns in young adults. The nature of barriers to help-seeking and the reduction in barriers following intervention may vary based on the nature of mental health concerns and the target age group.

Implications and future directions

The design of "ReachOut" enables easy implementation in resource-limited settings with minimal orientation and training. "ReachOut" merits further efficacy testing in controlled trials with a longer duration of follow-up and larger samples. Further studies may examine predictors of change in help-seeking inclination and behavior. Studies are needed to examine factors that may underlie the intention-behavior gap and improve actual help-seeking behavior rather than assessing only changes in attitudes and inclination. Sampling strategies that may help in enhancing the recruitment of male participants are worth examining. The intervention can be further fine-tuned and strengthened by increasing the number of tailor-made components over an extended implementation period, which may be useful for those requiring extended engagement to exhibit help-seeking behavior. Its potential for cost-effectiveness during large-scale implementation could be examined in subsequent studies. Intervention process and delivery guidelines can be manualized and made available in regional languages to standardize delivery and widen the reach of the intervention. Demonstrating the effectiveness of intervention delivery by lay counselors, non-mental health specialists, mentors and developing mechanisms for their training, support, and supervision as needed, can ensure scalability through the training of trainers.

## Conclusions

The present study is one of the first known attempts in India to test a simple technology-based intervention, "ReachOut," to improve help-seeking for common mental health concerns and provides preliminary evidence of its effectiveness, feasibility, and acceptability in distressed young adults. The targeted approach focusing on distressed non-treatment seekers used in the present study is crucial in early identification and timely treatment access to reduce the treatment gap and improve quality of life.
